# Comparison of Eating Habits in Obese and Non-obese Filipinas Living in an Urban Area of Japan

**DOI:** 10.1007/s10903-014-0024-9

**Published:** 2014-05-01

**Authors:** Chu Hyang Oh, Emiko Saito

**Affiliations:** Department of Nursing Sciences, Graduate School of Human Health Sciences, Tokyo Metropolitan University, 7-2-10 Higashiogu, Arakawa City, 116-8551 Tokyo Japan

**Keywords:** Filipinas living in Japan, Obesity, Eating habits, Acculturation

## Abstract

This study compares eating habits among obese and non-obese Filipinas living in an urban area of Japan. We used self-report questionnaires to study 635 Filipinos. Body mass index (BMI) and eating/lifestyle habits were noted. Obesity was defined as BMI ≥25 kg/m^2^. Seventeen percent (24/140) were obese. Results of the age-adjusted multiple logistic regression analysis show that the following responses were associated with obesity: “frequency of eating high green and yellow vegetables” (every day: 0, not every day: 1) [OR 4.9; 95 % confidence interval (CI) 1.6–14.8] and “frequency of eating high fruits” (every day: 0, not every day: 1) (OR .2; 95 % CI .1–.7). We suggest strategies to prevent obesity and improve eating habits among this Filipina population.

## Introduction

The number of registered foreign residents in Japan has been on the rise since the 1980s. For example, there were approximately 1.36 million foreign residents in Japan in 1995, and that number grew to 2.08 million in 2006, resulting in an increase of approximately 700,000 foreigners in just ten years. In 2006, foreign residents represented 1.6 % of the total population of Japan, and the country of origin for the largest group was Korea (North and South) (28.7 %), followed by China (26.9 %), Brazil (15.0 %), and the Philippines (9.3 %) [1]. The number of foreign residents is on the rise in Japan because of several factors, including a national labor shortage caused by a declining birthrate and an aging population. Globalization also plays a role. Japan’s economy has become increasingly global since the late 1980s, and more and more immigrants are consequently moving to Japan to seek job opportunities [2]. There is also a growing trend among short-term foreign workers to prolong their stay in Japan for various reasons [3].

Of all the foreign residents in Japan, the number of registered Filipino residents in particular is expected to rise in the next several years. This projected increase is related to several factors, including the economic partnership agreement (EPA) and the free trade agreement (FTA) as well as the increase in transnational marriages between Japanese men and Filipino women. In 2006, there were 193,488 registered foreign residents of Filipino nationality living in Japan, or 9.3 % of the total population of registered foreign residents [1]. In particular, Tokyo was home to 31,925 Filipinos in 2007, representing 8.3 % of the total population of registered foreign residents in the area [4].

In the Philippines, obesity has been a national health concern since the 1990s [5]. In recent years, the rise in obesity in the Philippines has been accompanied by an increase in the number of deaths attributable to lifestyle-related diseases. For example, increased BMI has been shown to be a risk factor for the development of hypertension [6–9]. In the Philippines, lifestyle-related diseases were the third-leading cause of death in 2000 [9], but Filipinos living abroad have also experienced problems with lifestyle-related diseases. In a study of Filipinos living in the USA, a lower rate of obesity was observed among Filipino women compared to Caucasian women [10]. However, this study also reported a higher prevalence of type two diabetes mellitus as a result of metabolic syndrome among Filipino women compared to Caucasian women [10]. While there is growing attention in the literature to obesity and lifestyle-related diseases among Filipinos living at home and abroad, we still know little about the health status and lifestyle habits of Filipinos living in Japan. This study addresses this empirical gap by investigating the eating habits of obese and non-obese Filipinas living in an urban area of Japan.

## Methods

### Design and Samples

We conducted this study between June and August 2007. The sample for this study included 635 Filipino participants that were recruited at six churches from an urban area in Japan. The participating churches regularly held a Mass attended only by Filipinos. On the day of the study, we distributed self-report questionnaires to all the participants who attended Mass and were able to participate in our study after Mass. Only Filipinas who attended this Mass in the church were enrolled in this study. After Mass, the subjects received the questionnaires and were verbally informed of the purpose of the study in English by the researcher. Correspondingly, the Filipino leader of the Mass attendance group also explained the purpose of the study in Tagalog. The subjects who participated in this study completed the distributed questionnaires immediately using a ballpoint pen provided by the researcher. When it was not possible to collect the completed questionnaires on the day they were distributed, we collected them the following week at the same church. The researcher verified the collected questionnaires with the Filipino group leader to assure the reliability of the responses. The questionnaires were in English and we attached a document providing a Tagalog translation. A bilingual Filipino researcher-collaborator translated the questions from English to Tagalog. The Ethical Committee of Tokyo Metropolitan University approved this study.

### Measures

We included the following demographic characteristics in the study: age, sex, medical history, subjective health status, level of work-related stress, educational level, occupation, financial situation, working hours per day, work hours during the day, types of co-habitants, length of stay in Japan, and Japanese language proficiency.

Participants’ self-reports of height and weight were used to calculate body mass index (BMI). The World Health Organization [11] has characterized BMI scores between 25 and 29.9 kg/m^2^ as pre-obese and BMI scores over 30 kg/m^2^ as obese. However, the World Health Organization and the International Task Force [12] consider a BMI ≥ 25 as obese in Asian people. Meanwhile, the Japan Society for the Study of Obesity has characterized BMI scores over 25 kg/m^2^ as obese [11, 13]. In this study, we categorized participants as obese if their BMI scores were over 25 kg/m^2^.

We also measured participants’ reported eating habits. The dietary composition of meals was assessed using the dietary variety score (DVS) developed by Kumagai et al. [14]. The DVS measures the consumption frequency of ten food groups (seafood, meats, egg, milk, soy products, green and yellow vegetables, seaweeds, fruits, potatoes, and oils and fats) over a period of 1 week. The distribution of the DVS scores ranges from 0 to 10, with higher scores representing higher food intake. The scores were calculated as follows: for each food group, ‘consume (almost) daily’ was assigned one point, and ‘consume once every 2 days,’ ‘consume once or twice a week,’ and ‘rarely eat’ were given no points. The points were then combined to produce a total score for each participant. We also used DVS scores to examine frequencies for the category of consumed foods (sweets and snacks and salty foods) and frequencies for the amounts of consumed food. The participants’ daily consumption patterns were also analyzed, including the number of meals per day, time spent per meal, frequency of eating breakfast, lunch and dinner over the course of 1 week, continuation of *merienda* habits, types of food eaten during *merienda*, and number of *meriendas* per day.

Participants’ reported lifestyle habits were also measured in this study using the cumulative lifestyle index (CLI) [15]. CLI scores are based on responses to six lifestyle questions: ‘sleeping at least 6 h a day’, ‘not smoking’, ‘eating breakfast at least five times a week’, ‘consuming snacks twice a week at most’, ‘no alcohol consumption’, and ‘exercising at least once a week’. The distribution of CLI scores ranges from 0 to 6.

### Analysis Procedure

The participants were divided into two groups based on their BMI scores. The ‘obese group’ had BMI scores ≥25 kg/m^2^, while the ‘non-obese group’ group had BMI scores <25 kg/m^2^. Eating and lifestyle habits were also compared between the two groups. We tested for significance between the two groups using the Chi square (*χ* ^2^) test, Fisher’s exact test, Student’s *t* test, and the Mann–Whitney *U* test. A significance level of <5 % was used, and statistical analyses were performed using SPSS 15.0 J (SPSS Japan Inc. Tokyo, Japan).

## Results

The number of collected questionnaires was 232 (37 %), and the number of valid responses was 140 (60 %). Among the 232 subjects who submitted the questionnaire, data from subjects 20 years of age or older who completed 80 % or more of the questionnaire and provided their height and weight were analyzed. Male subjects were excluded from this study. Demographic, lifestyle, and eating habit characteristics are listed in Table 1. The classification of weight by BMI in Filipinas is listed in Table 2. A mean age of 39.0 years (SD 8.9), participated in the study. Seventeen percent of the respondents (24/140) had BMI scores ≥25 kg/m^2^. In terms of age group, 88 % (21/140) of those with a BMI ≥25 kg/m^2^ were 40 years or older. The length of stay in Japan ranged from 1 month to 30 years, with a mean residency period of 10.6 years (SD 7.7). In terms of respondents’ occupations, 31 % (43/140) worked in manufacturing, 32 % (45/140) worked in service industries, and 16 % (23/140) was unemployed.

**Table 1:** Demographics, eating habits, and lifestyle of Filipinas living in an urban area of Japan (N = 140)

Characteristic	n (%) or mean ± SD
Average age (years)	39.0 ± 8.9
BMI (kg/m^2^)	21.8 ± 3.3
Medical history	
Hypertension	13 (9)
Diabetes	2 (1)
Subjective health status	
Healthy	116 (83)
Not healthy	16 (11)
Missing	8 (6)
Work-related stress	
Yes	86 (61)
No	31 (22)
Missing	23 (16)
Educational level	
College/university level or higher	89 (63)
High school level or lower	49 (35)
Missing	2 (1)
Occupation	
Unemployed	23 (16)
Employed	115 (82)
Missing	2 (1)
Financial status	
Above average	126 (90)
Poor	12 (9)
Missing	2 (1)
Co-habitants	
Spouse	79 (56)
Single	19 (14)
Length of stay (years)	10.6 ± 7.7
Japanese language proficiency	
Above average	98 (70)
Below average	42 (30)
*Eating habits*	
DVS (Max 10 points)	2.8 ± 1.6
Frequency of eating snacks	
Once or twice per week or less	80 (57)
Once every 2 days or more	57 (41)
Missing	3 (2)
Frequency of eating high salt content meals	
Once or twice per week or less	44 (31)
Once every 2 days or more	93 (66)
Missing	3 (2)
Frequency of consuming extreme amounts of food	
Once or twice per week or less	67 (48)
Once every 2 days or more	68 (49)
Missing	5 (4)
Average number of meals per day	3.0 ± 1.1
Time per meal (min)	20.2 ± 10.1
Continuing the habit of merienda	
Yes	96 (69)
No	41 (29)
Missing	3 (2)
No. of meriendas/day	1.1 ± 1.2
*Lifestyle habits*	
CLI (Max 6 points)	4.1 ± 1.5

**Table 2:** Classification of weight by BMI in Filipinas living in an urban area, Japan (n = 140)

BMI	<18.5	18.5–22.9	23–24.9	25–29.9	≥30
	Underweight	Normal range	At risk	Obese I	Obese II
	Non-obese			Obese	
	14(10)	83(59)	19(14)	21(15)	3(2)

Numbers are n (%)

Comparisons of the demographic characteristics between the obese and non-obese groups are shown in Table 3. The obese group had a significantly higher average age (*p* < .001) and a longer average length of stay in Japan (*p* = .01) than the non-obese group. The obese group had a significantly higher proportion of participants who were 40 years and over (*p* < .001), living in Japan for 5 years or longer (*p* = .01), living with hypertension (*p* = .02) and diabetes mellitus (*p* = .03).

**Table 3:** Comparison of demographic characteristics for obese and non-obese (n = 140)

Characteristic	Non-obese (n = 116)	Obese (n = 24)	*p*
Age (years)	37.8 ± 8.7	44.8 ± 7.3	<.001
Medical history			
Hypertension	7 (6)	6 (25)	<.001
Diabetes	0 (0)	2 (8)	.03
Subjective health status			
Healthy	96 (83)	20 (83)	.49
Not healthy	12 (10)	4 (17)	
Work-related stress			
Yes	73 (63)	13 (17)	.09
No	22 (19)	9 (38)	
Educational level			
Col./Univ. level or higher	71 (61)	18 (75)	.24
H.S. level or lower	43 (37)	6 (25)	
Occupation			
Unemployed	19 (16)	2 (8)	.53
Employed	91 (78)	21 (88)	
Financial status			
Above average	104 (90)	22 (92)	>.99
Poor	10 (9)	2 (8)	
Co-habitants			
Spouse	62 (47)	17 (50)	.18
Single	15 (11)	4 (12)	.75
Length of stay (years)	9.7 ± 7.6	14.8 ± 6.6	.01
Japanese language proficiency			
Above average	80 (69)	16 (67)	.64
Below average	32 (28)	8 (33)	

Numbers are mean ± SD or n (%). Statistical analyses: the Chi square (χ^2^) test was used for categorical variables; the Fisher’s exact test was used when an expected value was <5; Student’s *t* test was used for parametric continuous variables; and the Mann–Whitney *U* test was used for non-parametric continuous and ordinal variables. A significance level of *p* < .05 was used

Eating and lifestyle habits were also compared between the obese and non-obese groups. These findings are shown in Table 4. The obese group was significantly less likely to consume green and yellow vegetables (*p* = .03) and significantly more likely to consume fruits (*p* = .03) than the non-obese group. Taking into consideration the abovementioned results, variables where the responses differed significantly between the groups were examined to clarify the association with obesity by age-adjusted multiple logistic regression analysis (Table 5). We confirmed the correlation between the independent variables to check multicollinearity issue on the premise that we choose a variable. Thus, five of six variables were included in the multivariate logistic regression analysis. We omitted “length of stay” that had highly correlated with “age” (r = .455, *p* < .001) used as the control variable from the multivariate logistic regression analysis. Results show that the following responses were associated with obesity: “frequency of eating green and yellow vegetables” (every day: 0, not every day: 1) (OR 4.9; 95 % CI 1.6–14.8) and “frequency of eating fruits” (every day: 0, not every day: 1) (OR .2; 95 % CI .1–.7).

**Table 4:** Comparison of eating and lifestyle habits for the obese and non-obese (n = 140)

Characteristic	Non-obese (n = 116)	Obese (n = 24)	*p*
*Eating habits*			
DVS (Max: 10 points)	2.8 ± 1.7	3.0 ± 1.5	.66
Frequency of green and yellow vegetables			
Every day	66 (57)	8 (33)	.03
Not every day	47 (41)	16 (67)	
Frequency of eating fruits			
Every day	58 (50)	18 (75)	.03
Not every day	56 (48)	6 (25)	
Frequency of eating snacks			
1 or 2 per week or less	69 (60)	11 (46)	.17
1 every 2 days or more	44 (38)	13 (54)	
Frequency of eating high salt content meals			
1 or 2 per week or less	76 (66)	17 (71)	.73
1 every 2 days or more	37 (32)	7 (29)	
Frequency of consuming extreme amounts of food			
1 or 2 per week or less	57 (49)	10 (42)	.39
1 every 2 days or more	54 (47)	14 (58)	
Average no of meals/day	3.0 ± 1.1	2.8 ± .9	.57
Time per meal (min)	20.0 ± 10.0	21.3 ± 10.6	.47
Continuing the habit of merienda			
Yes	77 (66)	19 (79)	.28
No	36 (31)	5 (21)	
No. of meriendas/day	1.1 ± 1.3	1.1 ± .9	.42
*Lifestyle habits*			
CLI (Max 6 points)	4.1 ± 1.5	4.3 ± 1.7	.53

Numbers are mean ± SD or n (%). Statistical analyses: the Chi square (χ^2^) test was used for categorical variables; the Fisher’s exact test was used when an expected value was <5; Student’s *t* test was used for parametric continuous variables; and the Mann–Whitney *U* test was used for non-parametric continuous and ordinal variables. A significance level of *p* < .05 was used

**Table 5:** Factor associated with obesity (N = 140)

Variable	Odds ratio	95 % Confidence intervals
Frequency of eating green and yellow vegetables	4.9	1.6–14.8
Frequency of eating fruits	.2	.1–.7

Multivariate logistic regression analysis of factors associated with obesity: BMI ≥25: 1, non-obese: BMI <25: 0. Adjusted value: Age (40 years old or older: 1, under 40 years old: 0). Frequency of eating green and yellow vegetables (every day: 0, not every day: 1); Frequency of eating fruits (every day: 0, not every day: 1)

## Discussion

In terms of age, 45.3 % of Filipinas are between 30 and 39 years old [1]. The largest age group in our study was 40–49 years, which is roughly 11 years older than the national figures. Previous studies have investigated Filipino participants who have lived in Japan for 5 years or less [16–18]; however, in this study, the participants’ average length of stay in Japan was approximately 10 years. Similar to findings from previous studies [16, 17, 19], the highest level of education for most of the participants (approximately 60 %) in the present study was college, university, and above, and many worked in the manufacturing and service industries. Nearly 90 % of the participants in our study perceived their financial status to be above average. Almost all participants answered ‘healthy’ for the health status question; however, about 80 % reported work-related stress. These findings reveal that the Filipinas living in Japan who participated in our study have lived in Japan for a relatively long period of time, are mostly middle-aged, and generally perceive their financial status as above average.

Results showed that a significantly higher proportion of participants in the obese group were 40 years or older and had been living in Japan for five or more years. According to the Japanese National Nutrition Survey [20], 30.8 % of Japanese men and 24.6 % of Japanese women aged 40 years or older had a BMI ≥25 kg/m^2^. However, our study showed higher proportions of obesity in both men and women aged 40 years or older. While the obesity rate among Asian immigrants in the USA and Canada is often lower than that of the host country, studies show that obesity increases among immigrant populations with length of stay [21]. These findings highlight the importance of obesity prevention among Filipinas aged 40 years or older that have been living in Japan for five or more years.

Our study found that obese Filipinas living in Japan were more likely to suffer from diseases attributable to lifestyle habits, such as hypertension and diabetes mellitus. This situation is related to health studies of other immigrant populations in other countries. For example, Newbold and Danforth [22] and McKay et al. [23] found that the health status of immigrants in Canada is worse than that of their counterparts in the host population, and the prevalence of lifestyle-related diseases is significantly higher among immigrants. Similarly, Tashiro and Kuroyanagi [24] report a high prevalence of lifestyle-related diseases among Filipinas living in Japan. Moreover, the prevalence of both obesity and hypertension among Filipinos in the USA has been found to be higher than that of people in the Philippines [10]. In the Philippines, there is a high rate of people affected by hypertension, and the link between obesity and the development of hypertension has been well documented [6]. In the present study, many of the obese Filipinas in Japan already had lifestyle-related diseases. Measures are therefore needed help this population modify eating and lifestyle habits to reduce obesity and prevent disease progression. Obesity is a primary cause of lifestyle-related diseases, and its prevention will lead to the eventual prevention of such diseases.

The consumption of green and yellow vegetables every day and the daily consumption of fruits were associated with obesity. Although Higuchi [25] reported that Filipinas living in Japan cooked foods rich in vegetables, the results of our study showed that the proportion of individuals who consume green and yellow vegetables every day was significantly lower in the obese group. This contradiction may be partially explained by economic factors, as it has been reported that the poor living in an urban area of the Philippines tend to believe that convenience food is more wholesome than vegetables [26]. The present results show that the proportion of individuals who consume fruits every day was significantly higher in the obese group. This finding contradicts reports that fruit intake is heavily associated with a healthy diet [27]. However, it has also been reported that the excessive intake of fructose, which is abundant in fruit, increases visceral adiposity in overweight and obese adults [28]. Consequently, it may be necessary to advise this population to consume fruit in moderation. It is thought that the changes in eating habits in the Philippines may have influenced the eating habits of Filipinas living in Japan. The average dietary diversity score in the obese group was 3.0 points. A lower BMI is associated with the consumption of a variety of foods [29, 30]. It has also been reported that dietary diversity has decreased among obese women [31]. Therefore, Filipinas living in Japan also should be advised to consume a wide variety of nutritious foods to prevent obesity.

There are two effective health preventive approaches that emerge from these results. First, it is important to promote the improvement of eating and lifestyle habits for Filipinas in Japan and their families. These women provide care for babies and infants and can easily access administrative services and medical institutions. It is found that the proportion of obese mothers is significantly higher in obese children than non-obese children [32]. In our study, health promotion in mothers and children seems to be important because the proportion of mothers who live with their children is high. Administrative services and medical institutions are accessible, particularly for Filipinas in Japan who acquired Japanese nationality and a residency card by marrying a Japanese man. The following may be possible for such women: health-related information should be actively provided to them when they visit an institution for a medical examination or participate in an event; health guidance should focus on the family and consider the Filipino community living in Japan. Second, it is essential to obtain information on the social networks associated with institutions such as churches. Some institutions play an important role in ethnic networks and become places for the information exchange among its members, such as Filipinas living in Japan [33]. Additionally, through cooperation with such institutions, an effective approach would be to provide Filipinas living in Japan and gathering at these institutions with obesity prevention information or health guidance. Although it is difficult to perform direct intervention for Filipinas living in Japan who do not visit such institutions, we believe it is necessary to know the Filipino information network through “word of mouth”. The network should be promoted by establishing a close cooperative relationship with a support group to help Filipinas living in Japan.

There may be difficulties disseminating health-related information to Filipinas visiting Japan for the purpose of working. Thus, the establishment of good relationships with institutions such as churches or support groups that serve Filipinas living in Japan is important to effectively provide continuous service and achieve obesity prevention for Filipinas in Japan.

## Limitations and Conclusion

The participants in this study were Filipinas who lived in an urban area of Japan and attended churches affiliated with a foreign resident support group that assisted our study. Thus, the study captured only approximately 2 % of the total number of Filipinos registered as foreign residents in Tokyo. For Filipinos living in Japan, churches serve as an important place for building emotional and informational support networks. It is possible that Filipinos who do not use these networks may find it difficult to maintain good health. Future studies could focus on members of the Filipino community who do not attend church functions and events, thus providing a point of contrast to the results reported here. Additionally, BMI values in this study were calculated based on self-reported height and weight. However, future studies should obtain actual body measurements to ensure an accurate representation of participants’ health. The existing scale, dietary variety score (DVS), was used in this study as a tool to obtain information about the subjects’ eating habits. The DVS can mainly indicate the frequency of consumption of certain foods as a side dish, but not principal foods. Furthermore, the responses to the questions about eating habits were self-reported in this study. Therefore, in future studies, actual eating habits should be directly observed, including the intake of principal foods, in order to promote specific support for overall eating habits. Additionally, this study was a cross-sectional study. Therefore, in the future, a longitudinal study is required to examine the association between obesity and eating habits.

In this study, we conducted an investigation to clarify the prevalence of obesity and the associated eating habits among Filipinas living in an urban area of Japan. As a result, the following data were obtained, which suggest the necessity of promotion to prevent obesity and improve eating habits for Filipinas living in Japan:

1. The prevalence of obesity in Filipinas living in Japan was 17 %, which was lower than that in Filipino women living in the Philippines, but was higher than that in Japanese women 40 years or older of age.
2. A comparison of the obese and non-obese groups revealed that the average age and length of stay were significantly higher in the obese group, and that the prevalence of hypertension and diabetes were significantly higher in the obese group. Moreover, a comparison of the obese group and non-obese groups revealed that participants in the obese group were less likely to consume green and yellow vegetables every day, but more likely to consume fruits every day.
3. Compared with the non-obese group, the obese group had a significantly higher odds ratio of “frequency of eating green and yellow vegetables” and a significantly lower odds ratio of “frequency of eating fruits.”

These results suggest that intervention is necessary to prevent obesity and improve eating habits among Filipinas living in Japan.
